# The Book World of Medicine and Science

**Published:** 1903-02-07

**Authors:** 


### The Book World of Medicine and Science.

A Practical Handbook of Midwifery. By Francis W. Nicol Haultain, M.D., F.R.C.P.E. Second edition, entirely revised (illustrated). (London: The Scientific Press. Price 6s. net.)

The writer of this book is evidently a man of a methodical turn of mind who is able to picture to himself with considerable accuracy the relation which any one statement bears to other observations on the same subject. He has thus produced a book of extreme exactitude, one containing in a small compass all the facts bearing upon the art of midwifery, and one in which all these facts are set out in proper order and in due relation with each other. At the same time it must be confessed that it is not a very readable book, and such it probably was not intended to be. The book is, indeed, neither more nor less than a long and careful tabulation—a *catalogue raisonné*—of all that it is necessary for the practitioner to know on the subject. In this way it admirably fulfils the object for which it was written, namely, to be a practical help to the busy practitioner and at the same time to set before the student succinctly the chief points of importance in the study of obstetrics. It may, indeed, be regarded as an orderly note-book, in which each fact finds its proper place and is made to suggest the other facts which are related to it. Thus, to the practitioner it will act as a remembrancer, while to the student it will serve most usefully to give form and order and proper sequence to knowledge garnered bit by bit in the lecture-room, in the wards, or by the study of more diffuse treatises. It is not, however, an elementary handbook, for it enters quite as fully into complicated and instrumental as into ordinary cases, nor is it a book to be used as an introduction to midwifery by one who has access to no other sources of information on the subject.

Points of Practical Interest in Gynæcology. By H. Macnaughton-Jones, M.D., M.Ch., Q.U.I. Third edition, with 24 plates. (London: Baillière, Tindall and Cox. Price 4s. 6d. net.)

This is a more or less disconnected series of essays on gynæcological subjects. The subjects, however, are well chosen, being in every case matters of considerable interest, and they are so treated as to be full of information and of suggestions which may be useful even to specialists of considerable experience, for Dr. Macnaughton-Jones is not only a good teacher but a good learner, and while he speaks confidently from the standpoint of a large experience, he has not hesitated to add the lessons learned by visiting the cliniques of other operators and observing their methods and results. Thus it is an interesting and readable book. It is tainted perhaps with a degree of egotism which may offend the prurist, but after all a personal record must be personal, and without the "I," writ pretty large, the book would lose much of its flavour. Perhaps the best essay is that on affections of the female genitalia as factors in the production of neuroses and insanity. The discussion of the indications for operation in myoma is also well worth reading. Dr. Macnaughton-Jones is somewhat sarcastic in his remarks about the sort of "asepsis" which is indulged in by some surgeons. "It is obviously ridiculous," he says, "to see a surgeon who has prepared his hands making a resting-place for them on his hips, search in his waistcoat pockets for a knife or pencil, place them on the sides of a chair or stool, twirl his moustache, or use a pocket-handkerchief; but even worse acts than these have been noticed and commented upon by those who have visited some of our operation theatres." All of which is very amusing, but although, as everyone knows, many very odd things were done in the early days, asepsis as now generally practised in the operation theatres is hardly of the nature and quality suggested by the above paragraph.

The London Manual for 1903. Edited by Robert Donald. (London: Edward Lloyd. 1903. Price 1s. 6d.)

This most useful publication is both an annual and a manual. It is truly a manual in that it is a handbook to all that concerns public life in London, while its appearance as an annual ensures the up-to-dateness of the information it contains. London is so vast a place, its public life embraces so many phases, and the public bodies by which it is governed are so numerous, that some such guide and annual statement of the position of affairs becomes an absolute necessity, while even if only as a directory of the small army of permanent officials engaged in public work its publication would be thoroughly justified. But in addition to all this it contains a great deal of information in regard to many matters—lighting, tramways, tubes, education, waterworks, markets, hospitals, police, laws, politics, etc., which will be found not only useful but interesting reading.

Elements of Pharmacy, Materia Medica, and Therapeutics. By William Whitla, M.A., M.D. Eighth Edition. (London: Henry Renshaw. 1903.)

We welcome with great pleasure the issue of a new edition of Professor Whitla's ever-popular manual of materia medica. There are few books which have been so heartily accepted by a long series of students as this has been, and there must be very few books indeed which at the end of twenty years still so thoroughly meet the requirements of the student of the day as is done by this work in its present (the eighth) edition. The first part of the book is devoted to pharmacy—the compounding and dispensing of prescriptions—and here we find each operation thoroughly explained and illustrated by well executed drawings. Part II. describes the various modes in which medicines are administered, telling how prescriptions should be written; giving various rules as to Latin syntax which, so quickly does knowledge fade when unapplied, the student may already have forgotten; and arranging the various drugs in groups according to their actions. Part III. gives a full description of all the official remedies in the "British Pharmacopœia." Part IV. treats of therapeutics, describing the pharmacological and therapeutical action of all the official drugs; while Part V. is devoted to a somewhat less full description of the non-official remedies, poisons, and some other matters. The book has been brought thoroughly up to date, much of it has evidently been rewritten, and the whole will be found useful not only by students but also by those who are engaged in the active practice of their profession.

### BOOKS RECEIVED.

The Scientific Press.

"A Practical Handbook of Midwifery." By Francis W. Nicol Haultain, M.D., F.R.C.P.E. Second edition, entirely revised (illustrated).

Baillière, Tindall and Cox.

